# Evaluation of pulmonary artery pressure, blood indices, and myocardial microcirculation in rats returning from high altitude to moderate altitude

**DOI:** 10.1186/s41747-024-00514-5

**Published:** 2024-11-20

**Authors:** Chunlong Yan, Jinfeng Ma, Dengfeng Tian, Tingjun Yan, Chenhong Zhang, Fengjuan Zhang, Yuchun Zhao, Shihan Fu, Qiang Zhang, Mengxue Xia, Yue Li, Yanqiu Sun

**Affiliations:** 1 Department of Radiology, Beijing Anzhen Hospital, Capital Medical University, Beijing Institute of Heart Lung and Blood Vessel Diseases, Beijing, China; 2 Department of Radiology, Jining No.1 People’s Hospital, Jining, China; 3 Department of Radiology, Qinghai Provincial People’s Hospital, Xining, China; 4 Department of Hematology, Jining No.1 People’s Hospital, Jining, China; 5 Jining Medical College, Jining, China; 6 Graduate School of Qinghai University, Xining, China; 7 Department of Neurosurgery, Qinghai Provincial People’s Hospital, Xining, China

**Keywords:** Altitude, Hematocrit, Myocardial perfusion imaging, Pulmonary artery pressure, Rats (Sprague-Dawley)

## Abstract

**Background:**

To investigate changes in pulmonary artery pressure (PAP), blood indices, and myocardial microcirculation in rats returning from high altitude (HA) to moderate altitude (MA).

**Methods:**

Forty 4-week-old male Sprague-Dawley rats were randomly divided into four groups with ten rats in each group. One group was transported to the MA area (MA-group), and the other three groups were transported to HA (HA-group-A, HA-group-B, and HA-group-C). After 28 weeks of age, the rats from the HA area were transported to the MA area for 0 days, 10 days, and 20 days, respectively. PAP, routine blood tests, and computed tomography myocardial perfusion indices were measured.

**Results:**

Compared with the MA-group, the body weight of HA-groups decreased (*p* < 0.05), and PAP in HA-group-A and HA-group-B increased (*p* < 0.05). In the HA groups, PAP initially increased and then decreased. Compared with the MA-group, red blood cells (RBC), hemoglobin (HGB), and hematocrit (HCT) of rats in HA-group-A increased (*p* < 0.05). Compared with the HA-group-A, RBC, HGB, and HCT of HA-group-B gradually decreased (*p* < 0.05) while MCV decreased (*p* < 0.05), and PLT of HA-group-C increased (*p* < 0.05). Compared with the MA group, blood flow (BF) and blood volume (BV) of the HA-group-A decreased (*p* < 0.05). Compared with the HA-group-A, TTP increased first and then decreased (*p* < 0.05), and BF and BV increased gradually (*p* < 0.05). Pathological results showed that myocardial fiber arrangement was disordered, and cell space widened in the HA group.

**Conclusion:**

PAP, blood parameters, and myocardial microcirculation in rats returning from high to MA exhibited significant changes.

**Relevance statement:**

This study provides an experimental basis for understanding the physiological and pathological mechanisms during the process of deacclimatization to HA and offers new insights for the prevention and treatment of deacclimatization to HA syndrome.

**Key Points:**

- Forty rats were raised in a real plateau environment.
- Myocardial microcirculation was detected by CT myocardial perfusion imaging.
- The PAP of the unacclimated rats increased first and then decreased.
- The myocardial microcirculation of the deacclimated rats showed hyperperfusion changes.

**Graphical Abstract:**

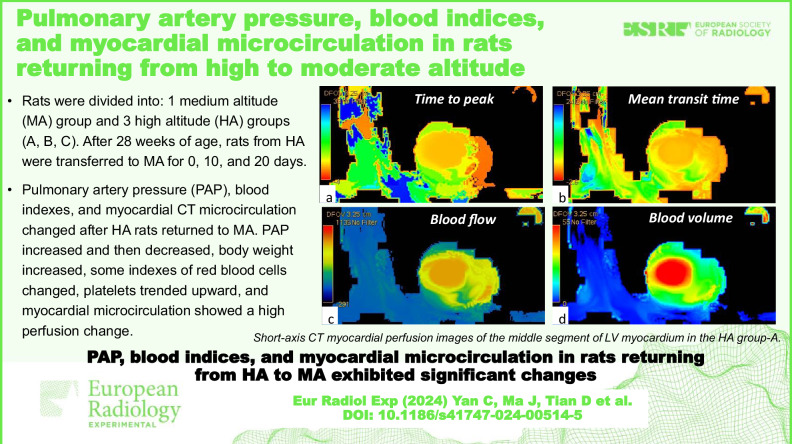

## Background

Deacclimatization to high altitude (DAHA) refers to a series of physiological and structural changes occurring in individuals after residing in a high-altitude (HA) region for an extended period. When they return to lower altitudes, the previously acquired acclimatization balance is disrupted. This transition leads to various physiological and pathophysiological adjustments as the body readapts to lower altitudes [1, 2]. Deacclimatization to high altitude syndrome (DAHAS) describes an overreaction to DAHA, resulting in a range of physical and even psychological disorders. These symptoms can include lethargy, forgetfulness, chest pain, anxiety, shortness of breath, headache, dizziness, fatigue, and inattention [3]. The duration and severity of DAHAS can vary. Milder cases may resolve without treatment, while severe cases significantly impact patients’ well-being, work, and daily life, necessitating symptomatic treatment [4, 5]. Unfortunately, there is limited research and global awareness regarding the prevention and treatment of DAHAS. With the rapid development of the economy and transportation in HA regions, increased cultural exchange, and population mobility between HA areas and lower altitudes, DAHA can no longer be overlooked. Therefore, studying DAHA is critically important for the health and well-being of HA populations.

In medical terms, HA is generally defined as an area located above 3,000 m above sea level, where noticeable biological effects occur due to reduced oxygen availability. However, some propose that the plateau altitude standard should be lowered to 2,500 m [6–8]. According to the altitude classification standard in Gerili’s “Plateau Medicine” [9], regions located at 500–1,500 m are considered low altitude, 1,500–2,500 m are moderate altitude (MA), 2,500–4,500 m are HA, 4,500–5,500 m are very HA, and altitudes above 5,500 m are classified as extremely HA. HA areas are characterized by low air pressure, reduced oxygen levels, low humidity, significant day-to-night temperature variations, and intense ultraviolet radiation, with low pressure and oxygen levels being the most prominent factors [6, 10].

Studies have shown that prolonged exposure to low-pressure and low-oxygen environments at HAs can adversely affect various bodily systems, particularly the cardiovascular system. This may result in structural and functional abnormalities in the heart, pulmonary artery remodeling, pulmonary hypertension, and disturbances in blood circulation [11, 12]. The body undergoes a series of adjustments upon returning from HA regions to lower altitudes, and currently, there is limited research on this topic, with unclear mechanisms explaining the associated pathophysiological changes.

This study investigated blood parameters, pulmonary artery pressure (PAP), and myocardial microcirculation through computed tomography (CT) myocardial perfusion imaging in rats returning from HA to MA areas, contributing an experimental foundation for understanding the mechanisms behind the pathophysiological changes associated with DAHA.

## Methods

### Animals

This study was approved by the Institutional Animal Research Committee of our local institute and conducted in accordance with the recommendations of the Guide for the Care and Use of Laboratory Animals by the Laboratory Animals Ethics Committee (approval no. 2010176A).

We selected 40 male Sprague-Dawley rats, aged 4 weeks, from Chengdu Dashuo Experimental Animal Company (Production license Number SCXK (Sichuan, China) 2020-030; License Number SYXK (Sichuan, China) 2018-119). The study was also approved by the Medical Ethics Committee of Qinghai Provincial People’s Hospital, and the handling of animals adhered to the “Guiding Opinions on Treating Laboratory Animals Kindly” issued by the Ministry of Science and Technology in 2006.

Rats were numbered using a number plate method affixed to their ears. They were divided into four groups: MA-group, HA-group-A, HA-group-B, and HA-group-C, each with ten rats. These rats were transported to the Xining area (MA, around 2,200 m) and Madao area (HA, around 4,300 m). The rats were raised until they reached 28 weeks of age and were then transported to Xining. Rats in HA-group-A stayed at MA for 0 days, HA-group-B for 10 days, and HA-group-C for 20 days, respectively.

Rats were raised in animal laboratories in Maduo and Xining areas. Indoor air circulation was maintained, natural light was provided to adjust the circadian rhythm, and the rat housing temperature was maintained between 18 °C and 25 °C with humidity levels of 40–60%. Adequate rat food was supplied daily.

### CT perfusion protocol

Before scanning, the rats were weighed, and abdominal anesthesia was administered using 10% chloral hydrate following the standard of 1–2 mL/100 g. Tail vein puncture was performed after successful anesthesia, and the rats were placed in a prone position on the examination bed.

A Revolution CT scanner (GE Healthcare, Pittsburgh, USA) was used for scanning. Scanning parameters included a tube voltage of 80 kV, tube current of 100 mA, 16-cm detector coverage, 5-mm layer thickness, a speed of 0.5 s/rotation. Iopromide (Haichang Pharmaceutical Co., LTD., Zhejiang, China) was used as the contrast agent. Each rat received 0.2 mL of contrast agent per 100 g of body weight via tail vein puncture, and cardiac perfusion images were obtained. Scanning procedure: the positioning phase (head to tail) was scanned first, and the contrast agent was injected at the beginning of the perfusion scan (manual injection was completed within 2–3 s according to the standard of 0.2 mL/100 g). The total scanning time was 50 s, the inflow period was 1 s, and the outflow period was 2 s. The scanning range included the head of the rat to the lower limbs, and the cardiac perfusion image was obtained.

**Fig. 1:**
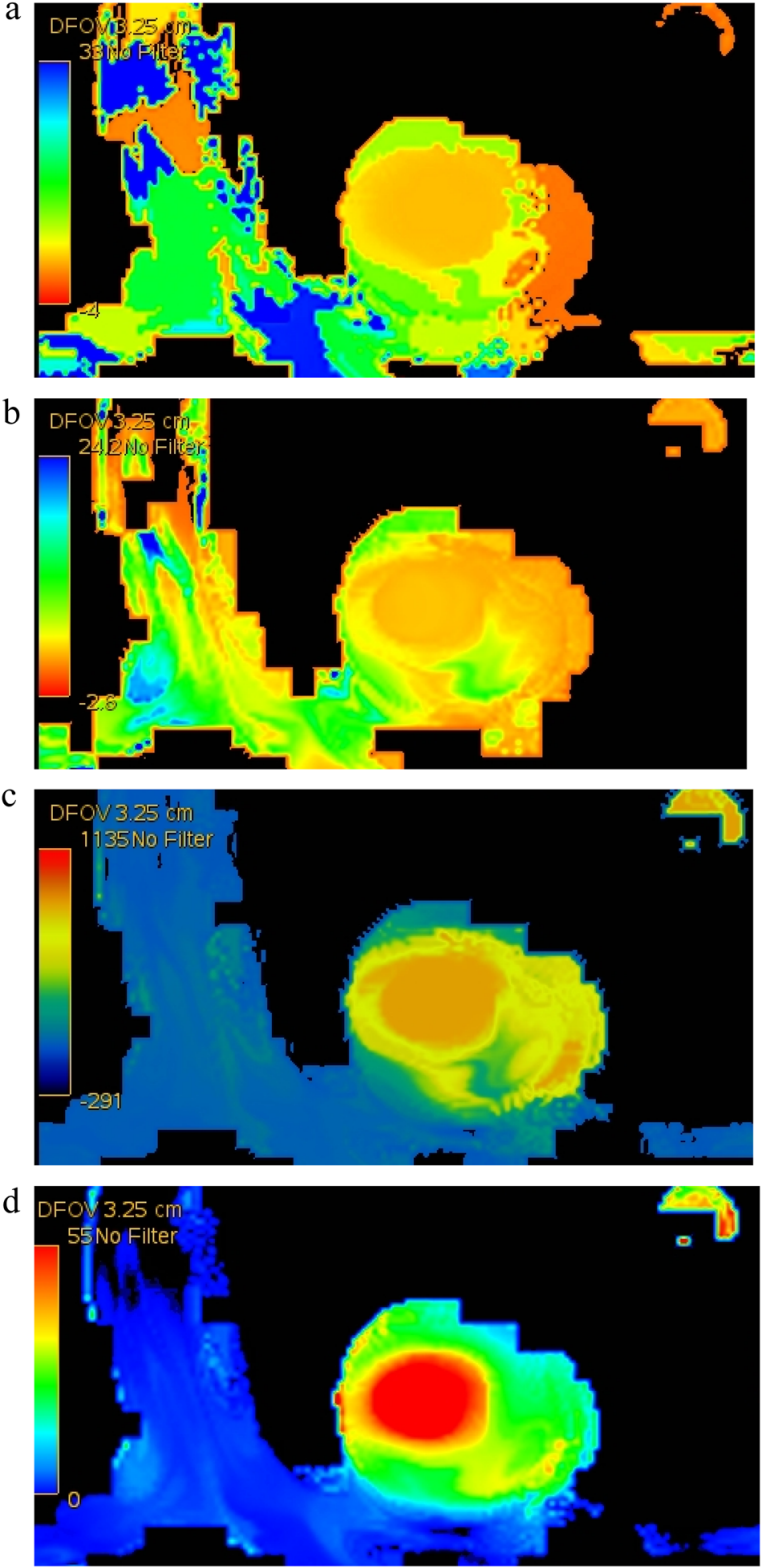
Short-axis CT myocardial perfusion images of the middle segment of the left ventricular myocardium in the high-altitude group A. **a** TTP image; **b** MTT image; **c** BF image; and (**d**) BV image

Perfusion images were imported into the GE AW4.6 workstation and entered into four-dimension perfusion software in CT tumor body protocol. Using the 17-segment heart segment analysis method developed by the American Heart Association [13], the perfusion image was adjusted to the left ventricular long axis and sagittal position. The left ventricular short axis position was reconstructed, and regions of interest were selected to generate time to peak (TTP), mean transit time (MTT), blood flow (BF), and blood volume (BV) values for each perfusion parameter (Fig. 1).

### PAP measurement and blood tests

After the CT scan, the rats were transported to the Plateau Medical Laboratory of Qinghai University. PAP was measured four times using a PowerLab biological data acquisition system (ADInstruments, Shanghai, China), and the average value was obtained. PowerLab is a high-performance biological signal processing device with four signal recording channels: pulse, blood pressure, electrocardiogram, and respiration, suitable for a variety of biological experiments to record and analyze multiple biological signals at the same time. The rats were placed on an operating table of moderate size and the limbs were fixed. PAP was measured by the right cardiac catheter method. First, the neck fur was smeared with wet gauze with normal saline to avoid the influence of rat hair. Then, the neck cortex of the rat was cut, the external jugular vein was separated, a hard plate was placed under the blood vessel, and the distal end was ligation. A small oblique incision was cut in the middle of the exposed blood vessel, and the catheter was slowly and gently inserted into the blood vessel. Waveform changes were observed in the monitor. When PAP waves appeared, four groups of data were recorded and average values were taken after the waveform stabilized.

The skin was completely exposed to the chest; the skin, muscles, and ribs of the rats were cut layer by layer, taking care not to damage the blood vessels around the heart. Abdominal aortic blood was collected for routine tests, including red blood cell count (RBC), hemoglobin (HGB), hematocrit (HCT), mean corpuscular volume (MCV), and platelet count (PLT).

### Pathology

Pathological samples were taken, and the myocardium’s pathological changes were observed with hematoxylin–eosin staining. The heart was separated by forceps at the aortic outlet of the heart, the blood in the ventricular cavity was rinsed with 0.9% sodium chloride solution, and the myocardial tissue was stored in 4% paraformaldehyde fixing solution for 24 h after drying with filter paper, and then dehydrated, impregnated with wax, and successively sliced (5 μm). The dehydration sequence of xylene was: (i) xylene; (ii) anhydrous ethanol; (iii) anhydrous ethanol—75% alcohol; and (iv) rinse with tap water. After hematoxylin staining for 3–5 min, the hydrochloric acid solution was differentiated and rinsed with tap water. Sections were dehydrated by gradient alcohol and stained with eosin. Then the slices were successively put into: (i) anhydrous ethanol; (ii) anhydrous ethanol; (iii) anhydrous ethanol-xylene; (iv) anhydrous ethanol-xylene; and (v) neutral gum tablets.

### Statistical analysis

Normal or near-normal data distribution was assessed through the Shapiro–Wilk test. Measurement data were expressed as mean ± standard deviation and one-way analysis of variance was used for comparison between groups. The least significant difference method was used for homogeneity of variances, and *p* < 0.05 was considered statistically significant. Data analysis was conducted using SPSS version 25.0 statistical software (Chicago, IL, USA).

## Results

**Fig. 2:**
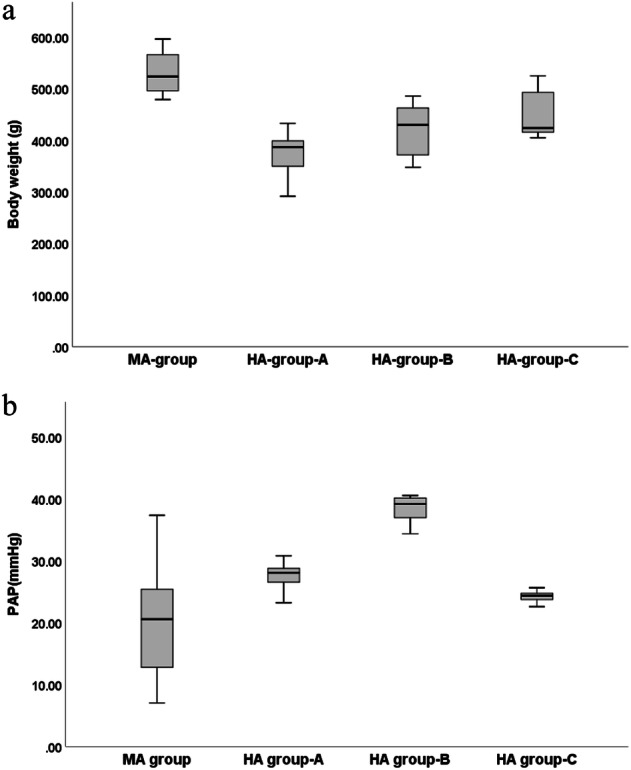
Boxplot of the index of body weight and PAP between MA-group, HA-group-A, HA-group-B, and HA-group-C. MA, Moderate altitude; HA, High altitude

Compared with the MA-group, the body weight of HA-group-A, HA-group-B, and HA-group-C was significantly decreased (*p* < 0.05) while the PAP of HA-group-A and HA-group-B was significantly increased (*p* < 0.05). In comparison with the HA-group-A, the body weight of HA-group-B and HA-group-C was significantly increased (*p* < 0.05), while the PAP of HA-group-B was significantly increased (*p* < 0.05) that of the HA-group-C showed a significant decrease (*p* < 0.05). Compared to HA-group-B, the PAP in HA-group-C was significantly decreased (*p* < 0.05) (Table 1 and Fig. 2).

**Table 1:** Comparison of body weight and PAP between MA-group and HA-group-A, HA-group-B and HA-group-C

Index/groups	MA-group, (*n* = 10)	HA-group-A, (*n* = 10)	HA-group-B, (*n* = 10)	HA-group-C, (*n* = 10)	*F* value	*p*-value
Body weight, (g)	533.80 ± 41.71	376.90 ± 39.93^Δ^	420.70 ± 50.50^Δ,*^	444.40 ± 43.51^Δ,*^	22.604	< 0.001
PAP, (mmHg)	19.97 ± 9.09	27.33 ± 2.45^Δ^	38.33 ± 2.15^Δ,*^	24.22 ± 0.92^¥^	26.204	< 0.001

Data are given as mean ± standard deviation

*PAP* Pulmonary artery pressure

^Δ^ *p* < 0.05 (compared with the MA-group)

^*^ *p* < 0.05 (compared with HA-group-A)

^¥^ *p* < 0.05 (compared with HA-group-B)

**Fig. 3:**
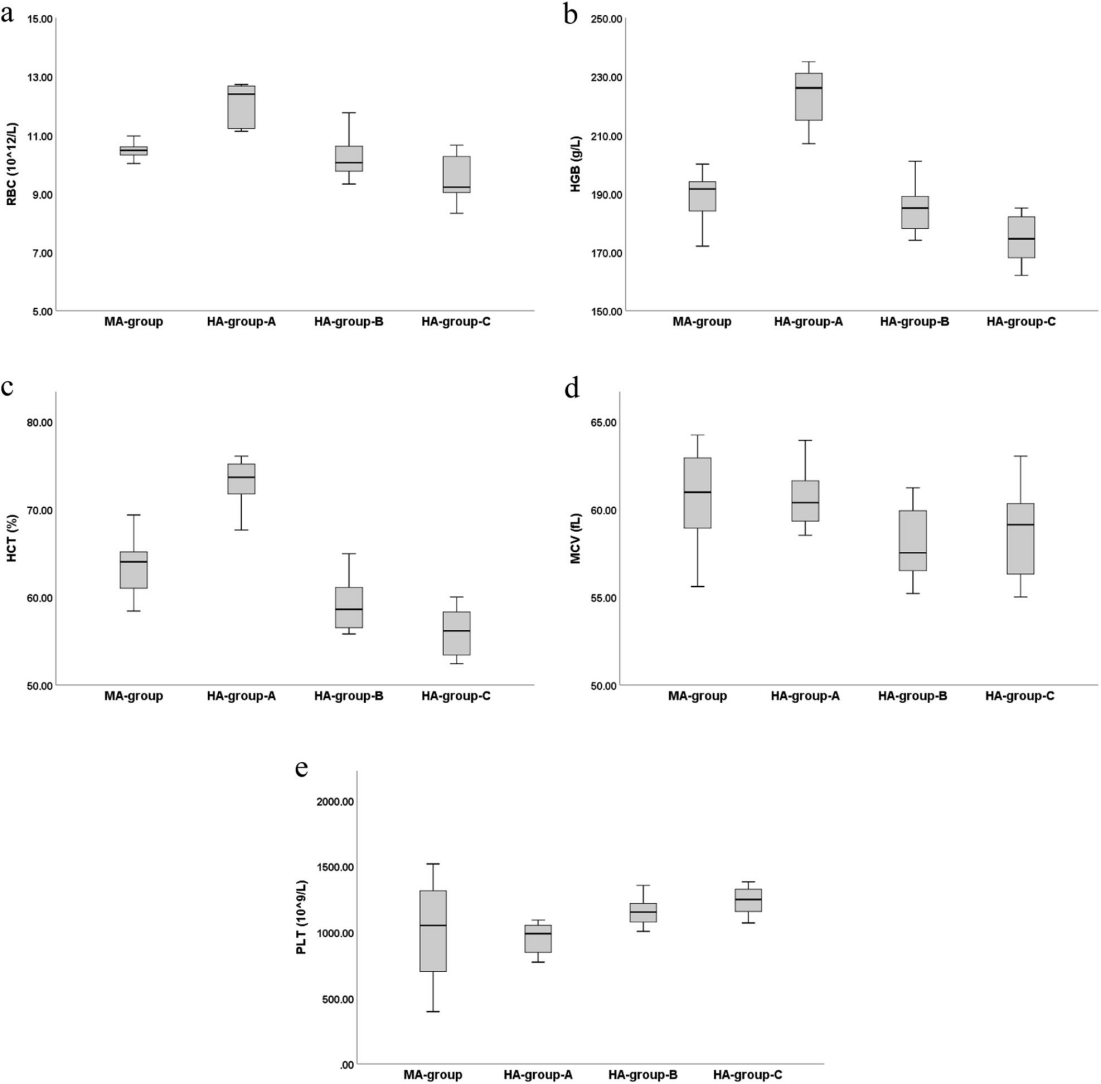
Boxplot of the index of blood indices between MA-group, HA-group-A, HA-group-B, and HA-group-C. MA, Moderate altitude; HA, High altitude; RBC, Red blood cell count; HGB, Hemoglobin; HCT, Hematocrit; MCV, Mean corpuscular volume; PLT, Platelet count

Compared with the MA-group, RBC, HGB, and HCT if the HA-group-A were significantly increased (*p* < 0.05), HCT and MCV of HA-group-B were significantly decreased (*p* < 0.05), RBC, HGB, and HCT of HA-group-C were significantly decreased (*p* < 0.05), while PLT of HA-group-C were significantly increased (*p* < 0.05). Compared with the HA-group-A, RBC, HGB, HCT, and MCV of HA-group-B were significantly decreased (*p* < 0.05), while RBC, HGB, and HCT in HA-group-C were significantly decreased (*p* < 0.05). Compared with the HA-group-B, RBC, and HGB, HCT in HA-group-C was significantly decreased (*p* < 0.05) (Table 2 and Fig. 3).

**Table 2:** Comparison of blood indices between MA-group and HA-group-A, HA-group-B and HA-group-C

Index/groups	MA-group, (*n* = 10)	HA-group-A, (*n* = 10)	HA-group-B, (*n* = 10)	HA-group-C, (*n* = 10)	*F* value	*p*-value
RBC, (10^12^/L)	10.48 ± 0.29	12.01 ± 0.71^Δ^	10.17 ± 0.71*	9.54 ± 0.80^Δ,*,¥^	25.362	< 0.001
HGB, (g/L)	189.70 ± 8.15	222.70 ± 9.97^Δ^	184.90 ± 8.08*	174.50 ± 7.63^Δ,*,¥^	59.955	< 0.001
HCT, (%)	63.70 ± 3.20	72.77 ± 2.86^Δ^	58.96 ± 2.88^Δ,*^	56.05 ± 2.69^Δ,*,¥^	63.046	< 0.001
MCV, (fL)	60.80 ± 2.56	60.68 ± 1.85	58.07 ± 2.16^Δ,*^	58.94 ± 2.82	3.180	0.036
PLT, (10^9^/L)	975.80 ± 383.76	949.30 ± 240.52	1,155.10 ± 112.08	1,267.60 ± 174.47^Δ,*^	3.685	0.021

Data are given as mean ± standard deviation

*RBC* Red blood cell count, *HGB* Hemoglobin, *HCT* Hematocrit, *MCV* Mean corpuscular volume, *PLT* Platelets count

^Δ^ *p* < 0.05 (compared with the MA-group)

^*^ *p* < 0.05 (compared with HA-group-A)

^¥^ *p* < 0.05 (compared with HA-group-B)

**Fig. 4:**
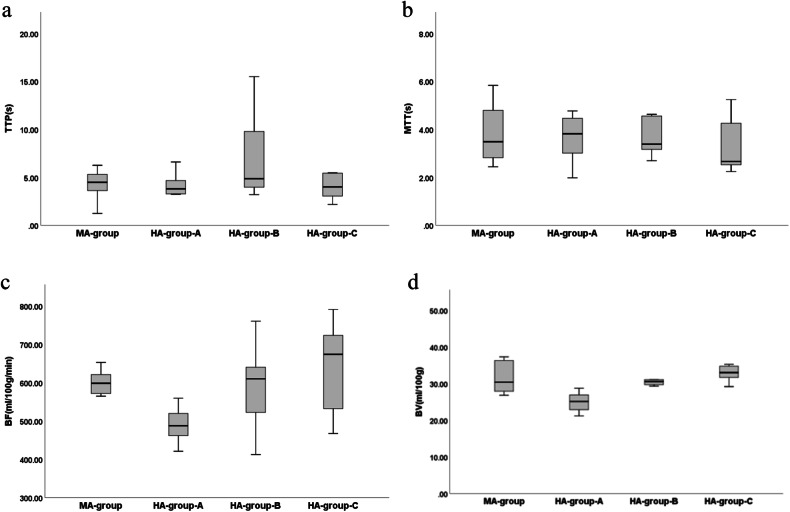
Boxplot of CT myocardial perfusion parameters between MA-group, HA-group-A, HA-group-B, and HA-group-C. MA, Moderate altitude; HA, High altitude; TTP, Time to peak; MTT, Mean transit Time; BF, Blood flow; BV, Blood volume

Compared with the MA-group, BF and BV in HA-group-A were significantly decreased (*p* < 0.05), while TTP in HA-group-B was significantly increased (*p* < 0.05) and BF of HA-group-C was significantly increased (*p* < 0.05). Compared with the HA-group-A, TTP, BF, and BV of HA-group-B were significantly increased (*p* < 0.05), while BF and BV in HA-group-C were significantly increased (*p* < 0.05). Compared with HA-group-B, TTP in HA-group-C was significantly decreased (*p* < 0.05) (Figs. 1 and 4).

**Fig. 5:**
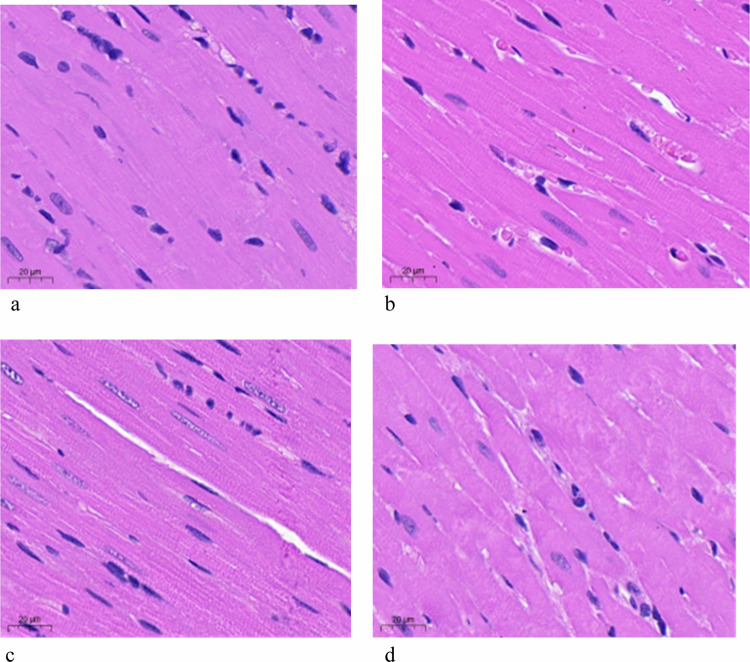
Hematoxylin and eosin staining pathology (× 400) of left ventricular myocardial fibers of MA-group (**a**), HA-group-A (**b**), HA-group-B (**c**), and HA-group-C (**d**). HA, High altitude; MA, Moderate altitude

At pathological examination, the myocardium in the MA group was clearly structured and the myocardium fibers were arranged neatly, while in the HA group, the myocardium fibers were arranged disordered, the cell volume increased, the space widened, and the interstitial inflammatory cell infiltration and fat cell infiltration increased (Fig. 5).

## Discussion

Our understanding of the pathogenesis of DAHA remains limited, and there has been insufficient research into the prevention and treatment of DAHAS, with this topic receiving relatively little attention on the global stage. As the economy, culture, and transportation in plateau regions continue to rapidly develop, and as population movement between plateau and lowland areas increases, plateau deacclimatization has emerged as a critical health concern [14]. Consequently, in-depth investigations into the physiological and pathological mechanisms underlying plateau deacclimatization hold great significance.

In recent years, the study of cardiovascular diseases has seen substantial progress, and clinicians have come to recognize the critical role of stable myocardial microcirculation in maintaining the heart’s normal physiological functions [15, 16]. Among the various imaging methods for assessing myocardial microcirculation, CT myocardial perfusion has gained increasing attention. Thanks to the rapid advancements in CT hardware and software, this imaging modality can provide a comprehensive evaluation of coronary artery conditions, cardiac anatomy, and myocardial perfusion in a single examination, offering valuable insights into both anatomy and function [17, 18].

Key parameters in CT myocardial perfusion imaging include: (BF through the vascular structures within a specific tissue); BV (the total volume of blood present within the vascular structures of a particular tissue); MTT (the time it takes for the time-density curve to drop to half of its maximum enhancement value at the beginning of contrast agent injection); and TTP (the time interval from the initiation of contrast agent injection to the point at which peak enhancement occurs). As research in this field advances, the insights derived from CT myocardial perfusion imaging are expected to play a significant role in the diagnosis and management of cardiovascular diseases [19, 20].

In this study, we found that the body weight, PAP, blood parameters, and myocardial microcirculation of rats returning from a high-altitude area to a moderate-altitude area had changed. The body weight showed a gradual increase trend, PAP increased significantly first and then decreased, some indexes of red blood cells and platelets had changed, TTP increased first and then decreased, and BF and BV showed a gradual increase trend.

These changes suggest that the body adjusts its blood composition to adapt to the changing oxygen environment at different altitudes. The body weight of rats in the HA area was lower than that in the MA area, and PAP, RBC, HGB, and HCT were higher than those in the MA area. After returning to the MA area for 10 days and 20 days, the body weight of rats in the HA area gradually increased, PAP first increased and then decreased, RBC, HGB, and HCT gradually decreased, and MCV was significantly reduced at 10 days. PLT increases significantly after 20 days of return. The reason may be that in the low-pressure and low-oxygen environment at the plateau, the partial pressure of oxygen in the alveolar decreases, the oxygen diffused into the blood of pulmonary capillaries decreases, and the partial pressure and saturation of arterial blood decrease accordingly. A long-term low-oxygen environment causes pathological remodeling of the pulmonary artery [21, 22], resulting in pulmonary hypertension. After returning to the MA, the atmospheric pressure in the environment increases. The oxygen content in the air increases, the partial pressure of oxygen in the alveoli increases, the oxygen diffused into the blood of pulmonary capillaries increases, and the oxygen saturation of arterial blood increases. In the early stage, there are relatively excessive RBCs in the blood to meet the oxygen supply of the body, which will be adjusted to achieve the homeostatic state of the internal environment. When the body returns to the MA area for 10 days, PAP increases, RBC decreases, HGB decreases, HCT decreases, and MCV decreases. When the body returns to the MA area for 20 days, PAP decreases. RBC, HGB, and HCT still decreased and PLT increased, suggesting that RBC, HGB, and HCT levels may take longer to fully recover.

As can be seen from Table 3, we found that the BF and BV of rats in the HA area were significantly lower than those in the MA area. TTP of rats in the HA area first increased and then decreased after returning to the MA area, BF and BV showed a gradually increasing trend, and BF and BV tended to be stable after 10 days of return. The reason may be that after the rats returned to the MA area, the oxygen content in the air increased, the arterial oxygen saturation increased, the number of red blood cells decreased, the blood viscosity decreased, the BF rate accelerated, the overall myocardial BV increased, TTP increased first and then decreased in the myocardial microcirculation, BF and BV showed a gradual increase trend, and BF and BV tended to be stable at 10 days after return.

**Table 3:** Comparison of CT myocardial perfusion parameters between MA-group, HA-group-A, HA-group-B, and HA-group-C

Index/groups	MA-group, (*n* = 10)	HA-group-A, (*n* = 10)	HA-group-B, (*n* = 10)	HA-group-C, (*n* = 10)	*F* value	*p*-value
TTP, (s)	4.33 ± 1.51	4.24 ± 1.25	6.71 ± 4.00^Δ,*^	4.03 ± 1.20^¥^	2.989	0.044
MTT, (s)	3.86 ± 1.15	3.72 ± 0.91	3.95 ± 1.37	3.21 ± 1.07	0.854	0.474
BF, (mL/100 g/min)	607.82 ± 46.28	490.36 ± 42.99^Δ^	592.94 ± 97.65*	641.50 ± 107.75^Δ,*^	6.747	0.001
BV, (mL/100 g)	31.50 ± 4.35	24.90 ± 2.49^Δ^	31.18 ± 4.46*	33.31 ± 3.19*	9.770	< 0.001

Data are given as mean ± standard deviation

*TTP* Time to peak, *MTT* Mean transit time, *BF* Blood flow, *BV* Blood volume

^Δ^ *p* < 0.05 (compared with the MA-group)

^*^ *p* < 0.05(compared with HA-group-A)

^¥^ *p* < 0.05 (compared with HA-group-B)

The study used rats that were returned from HA areas to MA areas. Future studies should explore transitions from different altitudinal environments, such as from sea level to plains to HAs. The study’s sample size was small, and the duration was relatively short. Expanding the sample size and extending the follow-up period would enhance the study’s robustness. In addition, there are differences between small mammals (rats) and large mammals (humans). All the rats in this study were male rats. In the future, the sample size will be increased to conduct further relevant studies on rats of different genders and large mammals.

In conclusion, PAP, blood indexes, and myocardial microcirculation were changed after HA rats returned to the MA region. PAP increased first and then decreased, body weight continued to increase, some indexes of red blood cells changed, PLT showed an upward trend, and myocardial microcirculation showed a high perfusion change. This study provides an experimental basis for the study of the physiological and pathological mechanism of the body during the process of DAHA, and provides new methods and ideas for the prevention and treatment of DAHA.

## Data Availability

The data that support the findings of this study are available from the corresponding author upon reasonable request.

## Abbreviations

BF: Blood flow
BV: Blood volume
CT: Computed tomography
DAHA: Deacclimatization to high altitude
DAHAS: Deacclimatization to high altitude syndrome
HCT: Hematocrit
HGB: Hemoglobin
MA: Moderate altitude
MCV: Mean corpuscular volume
MTT: Mean transit time
PAP: Pulmonary artery pressure
PLT: Platelets count
RBC: Red blood cell count
TTP: Time to peak
